# Theoretical Studies of IR and NMR Spectral Changes Induced by Sigma-Hole Hydrogen, Halogen, Chalcogen, Pnicogen, and Tetrel Bonds in a Model Protein Environment

**DOI:** 10.3390/molecules24183329

**Published:** 2019-09-12

**Authors:** Mariusz Michalczyk, Wiktor Zierkiewicz, Rafał Wysokiński, Steve Scheiner

**Affiliations:** 1 Faculty of Chemistry, Wrocław University of Science and Technology, Wybrzeże Wyspiańskiego 27, 50-370 Wrocław, Poland; mariusz.michalczyk@pwr.edu.pl (M.M.); rafal.wysokinski@pwr.edu.pl (R.W.); 2 Department of Chemistry and Biochemistry, Utah State University, Logan, UT 84322-0300, USA

**Keywords:** stretching frequency, NMR shielding, atomic charge, NBO, energy decomposition

## Abstract

Various types of σ-hole bond complexes were formed with FX, HFY, H_2_FZ, and H_3_FT (X = Cl, Br, I; Y = S, Se, Te; Z = P, As, Sb; T = Si, Ge, Sn) as Lewis acid. In order to examine their interactions with a protein, N-methylacetamide (NMA), a model of the peptide linkage was used as the base. These noncovalent bonds were compared by computational means with H-bonds formed by NMA with XH molecules (X = F, Cl, Br, I). In all cases, the A–F bond, which lies opposite the base and is responsible for the σ-hole on the A atom (A refers to the bridging atom), elongates and its stretching frequency undergoes a shift to the red with a band intensification, much as what occurs for the X–H bond in a H-bond (HB). Unlike the NMR shielding decrease seen in the bridging proton of a H-bond, the shielding of the bridging A atom is increased. The spectroscopic changes within NMA are similar for H-bonds and the other noncovalent bonds. The C=O bond of the amide is lengthened and its stretching frequency red-shifted and intensified. The amide II band shifts to higher frequency and undergoes a small band weakening. The NMR shielding of the O atom directly involved in the bond rises, whereas the C and N atoms both undergo a shielding decrease. The frequency shifts of the amide I and II bands of the base as well as the shielding changes of the three pertinent NMA atoms correlate well with the strength of the noncovalent bond.

## 1. Introduction

Our understanding of the H-bond (HB) represents a cornerstone of what has been learned over the years about solvation phenomena and the structure and function of biological systems [1,2,3,4]. The energetic and geometric aspects of the HB have raised our recognition of the requirements of a stable protein and the mechanism of countless enzymes. As part of the analysis of such systems, spectroscopic methods are commonly deployed to identify the presence of HBs, and to provide assessments of their strength. In particular, the shifts in certain IR bands or NMR peaks are frequently interpreted as a quantitative measure of the strength of each such bond [5,6,7]. As examples [3,8,9], the red shift of the A–H stretching frequency is thought to correlate with the strength of the AH···B HB, and there is a similar type of relationship for the downfield shift of the NMR peak of the bridging proton.

Recent years have witnessed a growing recognition of a set of newly rediscovered noncovalent bonds. Rather than utilizing a proton as a bridge between a pair of molecules, these related interactions incorporate a more electronegative atom from the right side of the periodic table. Although this bridging atom may have an overall partial negative charge, a detailed examination of its surrounding electrostatic potential reveals the existence of what has come to be called a σ-hole, a small region of positive potential lying directly along the extension of the covalent bond connecting it to the rest of the molecule. Depending upon the column of the periodic table from which this bridging atom is drawn, the resulting noncovalent bond with a nucleophile is designated as a halogen (XB), chalcogen (YB), pnicogen (ZB), or tetrel (TB) bond. Like the HB, this class of bonds [10,11,12,13,14,15,16,17,18,19,20,21,22,23,24,25,26,27,28,29,30,31,32,33,34,35,36] are similarly derived from a primary electrostatic attraction, supplemented by substantial amounts of charge transfer, polarization, and dispersion components.

These noncovalent bonds have a number of features in common. They are of a strength comparable to or even greater than a HB. Each such bond is strengthened by electron-withdrawing substituents which intensify the σ-hole. These bonds are usually strengthened as one moves down each column of the periodic table, for example, Cl < Br < I. First-row atoms (i.e., F, O, N, and C) engage in only weak bonds of this type if at all, but can be coaxed into measurable interactions by appropriate substituents or by adding a charge [37,38,39,40,41,42,43].

Despite the recent amassing of fundamental information about these noncovalent bonds, their effects upon the IR and NMR spectra have attracted far less attention. The data that have appeared [37,44,45,46,47,48,49,50,51,52,53,54] are informative to be sure, but they do not consider these systems in a systematic manner. As such, a thorough understanding is not available of the manner in which each type of interaction modulates the spectra, or of the processes by which they might do so. Such information would be essential in detecting their presence in a given chemical or biological system. It would also be especially useful if correlations could be established, between certain spectroscopic parameters and the strength or geometry of a given bond, as has proven so very useful for HBs over the years.

The present work represents an attempt to remedy this lack of information. The focus is placed here on proteins where such noncovalent bonds may be an important component in their structure and function. Indeed, sparked by very recent recognition of the importance of such interactions, researchers have begun to search for their presence in previously solved structures and have successfully identified them in a growing number of cases [18,55,56,57,58,59,60,61,62]. That work would be greatly facilitated if one knew the trademark spectroscopic fingerprint with which to search for such bonds.

A number of model systems were generated in which a halogen, chalcogen, pnicogen, or tetrel bond was present. Their interactions with a protein were simulated by using a model of the peptide group as electron donor, in much the same way that the peptide O atom acts as proton acceptor in so many protein HBs. Quantum calculations evaluated the strength of each interaction, as well as its geometric properties. The effects of each bond upon the IR and NMR spectra of the system were determined and compared with the binding strength, identity of the bond, and nature of the specific bridging atom. In this way, a systematic set of rules was generated that may hopefully assist in identifying these types of bonds in a complicated system, and providing some measure of their strength.

As the goal of this work was to determine how the spectroscopic features of H-bonded systems compared with the same aspects of related noncovalent bonds, a variety of different Lewis acids (i.e., σ-hole donors) were chosen to cover a broad range in each category. The full range of hydrogen halides, namely, FH, ClH, BrH, and IH, represent molecules of varying acidity that donate a proton in a HB. Within the set of halogen-bonding molecules, FCl, FBr, and FI all place an electron-withdrawing F substituent on the halogen atom so as to create a σ-hole which can engage with a nucleophile. A similar philosophy was adopted for the other types of Lewis acids. HFS, HFSe, and HFTe cover a range of chalcogen-bonding molecules. Pnicogen-bonding was invoked via H_2_FP, H_2_FAs, and H_2_FSb, whereas H_3_FSi, H_3_FGe, and H_3_FSn represent the set of tetrel-bonding molecules. Of course, one would not expect these molecules themselves to typically be present within a protein environment, as they serve merely as models of larger molecules. With respect to nucleophile, the N-methylacetamide (NMA) unit serves as a commonly invoked model of the protein peptide group. Its O atom is expected to form not only HBs with proton donors, but also the other types of noncovalent bonds with the various Lewis acids. It was hoped that this choice of base would enable parallels to be drawn to the spectroscopic aspects of these interactions within the confines of a protein.

## 2. Results

**Figure 1:**
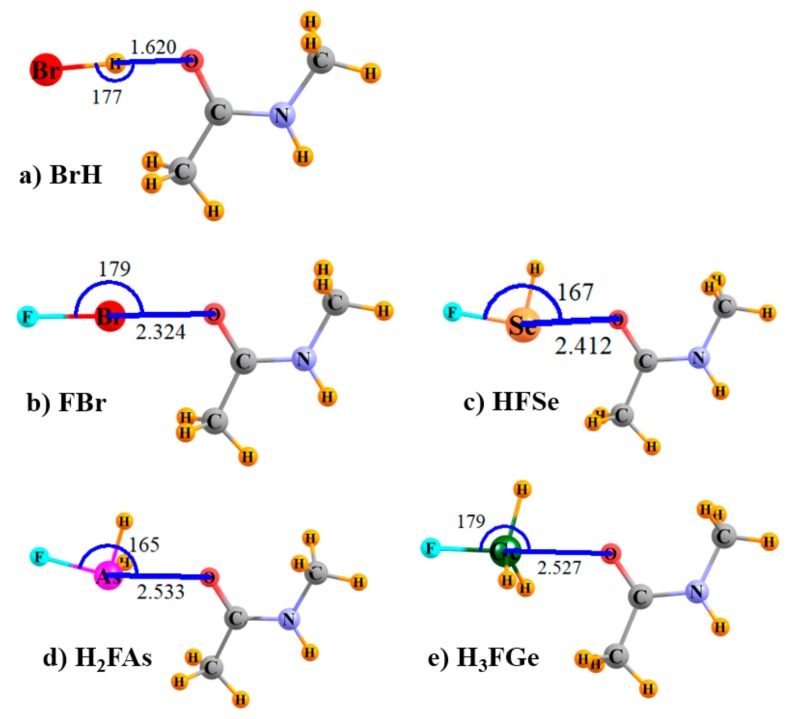
Optimized geometries of indicated Lewis acids with N-methylacetamide (NMA). Distances in Å, angles in degrees.

The optimized geometries of some of the sample systems are displayed in Figure 1; others are quite similar. Coordinates of all optimized complexes are available in the Supplementary Materials section. It may be noted that the relative orientations are as would be expected for each of the types of bonds under consideration here, commensurate with the positions of the σ-holes on each type of Lewis acid, and with the O atom of NMA serving as the electron donor atom.

### 2.1. IR Spectra

The first four rows of Table 1 reiterate the types of changes in the monomers that are well known for HBs. The X–H bond of the proton donor is elongated and its stretching band is shifted to the red and intensified. In these particular cases, the stretch of the XH bond increases from 0.032 Å for the smallest X=F atom up to 0.069 Å for the largest X=I. The red shift is equal to approximately 600–700 cm^−1^ for the entire range of X atoms. The intensification of the band is expressed as the ratio of the intensity within the complex to that within the HX monomer. This quantity is 14 for HF and increases up to 147 for HBr, and then makes a large jump to nearly 48,000 for HI. The changes occurring within the proton acceptor NMA molecule are somewhat smaller but still easily detectable. Upon forming a HB with each HX molecule, the C=O bond of NMA elongates by 0.01 Å. Its stretching frequency, commonly known as the amide I band, shifts to the red by 30–170 cm^−1^, and becomes more intense by a factor of 1.6–10. In each case, the magnitude rises along with the size of the X atom, that is, F < Cl < Br < I. The amide II band is a combination of C–N stretch and N–H bend. This mode shifts to the blue by 25 cm^−1^, with little sensitivity to the identity of X. There is a small diminution of its intensity for the three lighter X atoms, and an intensification for HI.

The lower portions of Table 1 refer to halogen, chalcogen, pnicogen, and tetrel bonding, abbreviated as XB, YB, ZB, and TB, respectively. The general patterns for all of these types of noncovalent bonds echo the changes observed for HBs. The bond within the Lewis acid molecule that engages with the partner NMA elongates, and its stretching mode shifts to the red and becomes more intense. Just as in the case of HBs, the C=O bond of NMA also becomes longer, shifts to the red, and intensifies. The changes induced in the amide II band are also quite similar to those caused by HB formation, and of comparable magnitude.

Systematic patterns also occur in the data. The magnitudes of the changes within the σ-hole donor follow the general decreasing order of XB > YB > ZB ~ TB. The bond elongations generally increase along with a larger atom (e.g., P < As < Sb), whereas the spectroscopic changes follow an opposite pattern, diminishing with a larger atom. While the bond stretches are roughly of the same magnitude as observed in the HBs, the spectroscopic data are much smaller. For example, the formation of a HB by ClH shifts its stretching frequency by 700 cm^−1^, and intensifies the band by a factor of 50, whereas the FCl bond is red-shifted by only 100 cm^−1^, and intensifies by 10-fold.

With regard to the NMA electron donor, the perturbations also follow the XB > YB > ZB ~ TB order. Within any given class of bond (e.g., chalcogen), all parameters, such as bond stretch, red shift, and intensification, grow in magnitude as the atom in question becomes larger (e.g., S < Se < Te). These perturbations are comparable to those noted as a result of HB formation. In summary, the IR perturbations to the spectra of both electron acceptor and donor molecules within any of these noncovalent bonds are quite similar to those expected for HBs, notwithstanding the larger changes in the HB proton donor′s stretching frequency, which are due in part to the much lighter mass of the bridging proton.

### 2.2. NMR Spectra and Atomic Charges

Tables S1 and S2 (Supplementary Materials) display the NMR isotropic chemical shielding of each of the unperturbed monomers, and the analogous data for the optimized complexes are contained in Table S3 (Supplementary Materials). More importantly, Table 2 lists the changes in the NMR isotropic chemical shielding of the various relevant atoms resulting from the formation of each of the noncovalent bonds. The topmost section of data reflects the deshielding that occurs on the bridging proton as a result of HB formation. This deshielding varies from 7 ppm for FH up to 11 ppm for BrH and IH. In contrast, the other bridging atoms, whether X, Y, Z, or T, all undergo an increase in their shielding. The magnitude of this change is largest for X, then decreases in the order of Y > Z > T. For each class of bond, the shielding increase rises dramatically along with the size of the atom. For example, the Cl, Br, and I atoms of the XB complexes increase their shielding by 239, 929, and 2197 ppm, respectively.

The F atom bound to the bridging atom suffers a loss in shielding, also in the same X > Y > Z > T order. (The Br and I atoms of BrH and IH increase their shielding upon HB formation.) The O atom of the NMA C=O bond raises its shielding, whereas its C and N neighbors suffer a drop. Probably owing to its direct involvement in each noncovalent bond, the O increases are larger in magnitude than the decreases observed in C and N. The O increases generally fall into the familiar X > Y > Z > T pattern. While the O shielding increase diminishes with larger X atom in the XH⋯NMA complexes, its increase rises regularly along with larger bridging atom size in the other noncovalent bonds. In contrast, the C and N shielding changes show lesser sensitivity to bond type or to bridging atom size. Importantly, the general patterns on all three NMA atoms for the HBs are repeated for the other noncovalent bonds.

Certain points of consistency between the NMR shielding patterns and the changes in natural atomic charge accompany the formation of each of these interactions, as reported in Table 3. Considering first the NMA electron donor molecule, the O atom generally acquires additional electron density, which is consonant with its enhanced shielding. The drops in the C and N shielding can likewise be connected to the reduced negative charges of these atoms. The bridging atoms of the various Lewis acid molecules acquire a larger negative charge as a result of complexation, which is consistent with their increased shielding. On the other hand, the quantitative relationships are not correlated. For example, while the shielding increase of the bridging atoms rises along with the size of this atom, the increased negative charge varies in the opposite direction, that is, smallest for larger atoms. A perhaps even more fundamental discrepancy occurs for the F atoms. Even though each such atom acquires additional negative charge from the complexation process, its shielding drops by a large amount.

**Figure 2:**
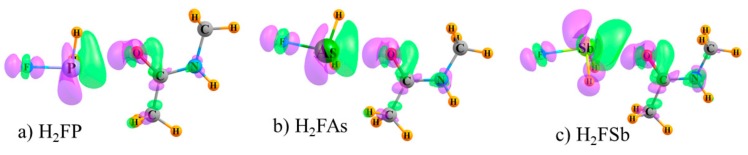
Regions of density gain (purple) and loss (green) in complexes pairing NMA with (**a**) H2FP, (**b**) H2FAs, and (**c**) H2FSb. Contour shown is ±0.0015 au.

Of course, the density changes that occur as a result of complexation are more complex than can be fully encapsulated by a simple numerical atomic charge. The actual density shifts are shown as a full three-dimensional map in Figure 2 where increases are shown in purple and losses in green. The systems illustrated in Figure 2 represent the three ZB systems, but the diagrams for the XB, YB, and TB bonds are quite similar. (The electron redistribution patterns of all systems are displayed in Figure S1 (Supplementary Materials) at two different contour levels, Δρ = 0.0005 and 0.0015 au.) It is clear that there are regions of both gain and depletion surrounding each nucleus, and the atomic charge data represent only a broad summation of both effects. For example, while Table 3 indicates an overall increase of electron density on the F atom, the more detailed examination in Figure 2 shows a region of loss at the F nucleus, with areas of gain along the F–Z axis. Of course, the presence of both gain and loss regions does not imply full cancellation, so the charge changes in Table 3 are not zero. It may be concluded that the depletion close to the nucleus is the major factor responsible for the reduction in the NMR shielding. The Z atom loses density on the side toward the NMA O atom, but compensates with a gain along the Z–F bond. According to Table 3, the green area of loss predominates for Sb, and the opposite occurs for the smaller P and As, which is consistent with the sizes of the lobes in Figure 2, although of course it is difficult to quantify the totals from merely overall lobe size. As for the NMA molecule, all three atoms of interest are centers of both purple and green lobes. However, it is the green depletion lobes which are closest to the C and N nuclei which help explain their shielding reduction, whereas the O nucleus lies within a purple density gain region, larger than the surrounding green area, and so this results in a shielding increase.

### 2.3. Relation to Binding Energies

One may expect some correlation between the spectroscopic aspects of these interactions and their energetic strength. The interaction energies of each complex are displayed in Table 4 at three different levels of theory. While the BLYP-D3/Def2TZVPP binding energies (II) are somewhat larger than the two ab initio levels, it is gratifying that the MP2 results in column I are quite similar to the CCSD(T) data in column III, both with the same aug-cc-pVDZ basis set.

**Figure 3:**
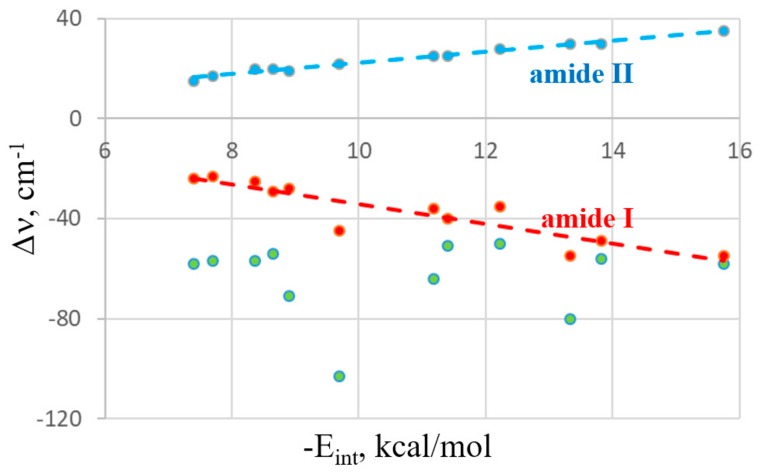
Relationship between MP2/aug-cc-pVDZ interaction energy and changes in frequencies of F–X stretch (green points), amide I (red), and amide II (blue). Broken lines indicate linear fits.

As is typically the case in these types of bonds, the interaction grows along with the size of the bridging atom, whether X, Y, Z, or T. HB energies are directly related to the acidity of the proton donor and therefore follow the pattern of HF > HCl > HBr > HI. The relationship between the interaction energies and the frequency shifts of the various bands are exhibited in Figure 3. The data presented exclude the HB systems due to their somewhat anomalous behavior with respect to the other noncovalent bonds. For example, HB energy lessens as the X atom of XH grows larger, whereas XB behaves in the opposite fashion. The green data points refer to the Δν band shifts of the F−A bond of the Lewis acid (A refers to any of the bridging atoms). There is much scatter and one can conclude that only a poor correlation occurs. Indeed, the correlation coefficient between this frequency shift and the interaction energy is only 0.04. The two points that are furthest from any linear correlation with the largest red shift correspond to the FCl and FBr halogen bonds. However, even if these two outliers are removed, the correlation remains poor, with a correlation coefficient of 0.10. The shifts of the NMA bands are another story, as they are nearly linear functions of the energetics. The red line in Figure 3 relates to the amide I band, whose correlation coefficient with E_int_ is 0.90. Even better is the blue line representing the amide II band with a correlation coefficient of 0.99. It thus appears that the frequency shifts occurring within the electron donor molecule serve as an excellent indicator of the bond strength in these systems.

**Figure 4:**
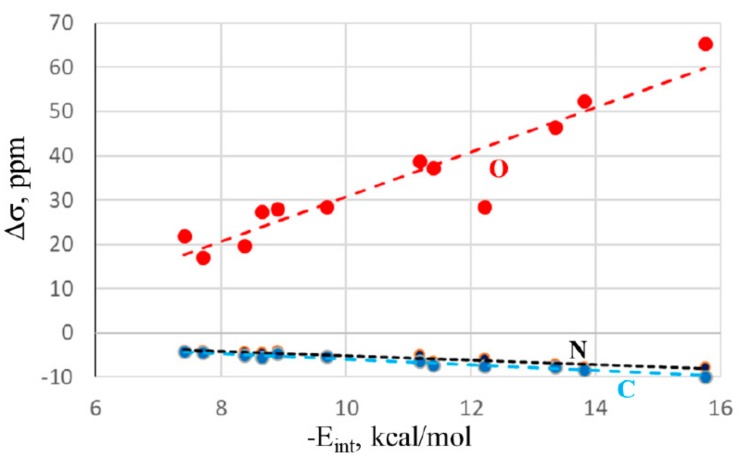
Relationship between MP2/aug-cc-pVDZ interaction energy and changes in NMR shielding of indicated atoms of NMA. Broken lines indicate linear fits.

There is also a certain correspondence between the energetics and NMR data. Figure 4 shows a nearly linear relationship with the changes in the NMR chemical shifts of the O, C, and N atoms of the NMA electron donor molecule. The correlation coefficients are 0.94, 0.95, and 0.98, respectively. Again, adding the HB systems to these data sets deteriorates the correlations, dropping them down below 0.90. The shifts of the bridging X atom do not correlate well at all, as is evident from the large fluctuations in the first column of Table 2. Thus, along with the IR shifts occurring within the electron acceptor, the NMR chemical shifts can also act as a valuable indicator of bond strength.

### 2.4. Contributions to Binding Energies

It is well known that electrostatic attraction in noncovalent bonds such as these is supplemented by orbital interactions and dispersion energy. A decomposition of the total interaction energies into its constituent parts was carried out and the results are reported in Table 5. There are similarities between all of these bonds first in that electrostatics account for at least half of the total interaction. This percentage contribution rises with the size of the bridging atom, with the exception of HBs where the opposite trend is noted. Orbital interactions, which include internal polarization and charge transfer, make up a bit less, approximately in the range of 31–47%, and their percentage contribution tends to diminish with larger bridging atoms. Dispersion plays a lesser role, contributing 10% or less. It is interesting that the dispersion′s percentage contribution follows the approximate pattern of XB < YB < ZB ~ TB.

Within the realm of the charge transfer, NBO offers a means of examining this phenomenon in more detail. The first column of Table 6 displays the energetic consequence of charge transfer from the lone pairs of the NMA O atom into the relevant σ* antibonding orbital of the Lewis acid, that is, σ*(HX) for HB and σ*(AF) for the others. These quantities are largest for the HB systems, and then diminish in the order of XB > YB > ZB > TB. Within each category, there is a general increasing trend with the size of the A atom, with some exceptions. A broader view of the charge transfer sums all of the interorbital interactions from base to acid. As seen in the next column of Table 6, these quantities are only slightly larger but follow similar trends. Rather than consider the energies of each transfer, one can simply evaluate the total charge transferred from base to acid. This quantity, designated as CT in Table 6, also follows the XB > YB > ZB > TB trend, but in this case, the HB values are smaller than those computed for XB and YB.

The strengths of these intermolecular bonds can also be measured via AIM analysis of the electron density topology. Some of the most common indicators are reported in Table S4 (Supplementary Materials). As is the case with the other measures described above, AIM verifies the general HB > XB > YB > ZB > TB trend, as well as a tendency for stronger bonds to be associated with larger bridging A atoms.

## 3. Computational Methods

Full optimizations of isolated monomers and their complexes with NMA were performed at the MP2/aug-cc-pVDZ level of theory [63,64] (coordinates of obtained complexes are given in Table S5). For accurate electronic descriptions of the heavy I, Te, Sb, and Sn atoms, the aug-cc-pVDZ-PP basis set with pseudopotentials that include relativistic effects was taken from the EMSL library [65,66]. Frequency analysis confirmed these structures as true minima. The DFT BLYP-D3/Def2TZVPP [67,68] method was utilized in the NBO analysis (preceded by full geometry optimizations) so as to include electron correlation. CCSD(T)/aug-cc-pVDZ [69,70] single-point calculations, using MP2 geometries, provided a check on the MP2 energetics. Nuclear magnetic resonance (NMR) chemical shieldings were computed via the GIAO approach at the MP2 level. To provide full flexibility to the inner-shell electrons for purposes of NMR calculations, the all-electron Sapporo-DKH3-DZP-2012-diffuse [71] basis set, with relativistic effects included, was used for 4th row atoms, instead of the effective core potential of aug-cc-pVDZ-PP. Computations were carried out within the framework of the Gaussian09 suite of programs [72].

For isolated monomers, the maxima of molecular electrostatic potential (MEP) were located and visualized using MultiWFN and WFA-SAS programs on the 0.001 au electron density isosurface at the MP2 level [73,74,75]. Interaction energies of dimers were assessed as the difference in energy between the complex and the two monomers in their distorted geometry within the complex. The basis set superposition error (BSSE) was removed via the standard counterpoise procedure [76]. QTAIM analysis was employed to identify and quantify specific interconnections between monomers, using AIMAll Professional software [77]. NBO analysis (GenNBO 5.0) [78] was applied in order to measure charge transfers between orbitals and to compare natural charges before and after complexation. Decomposition of interaction energies was performed using the Morokuma–Ziegler EDA scheme implemented in the ADF program at the BLYP-D3/ZORA/TZ2P level [79,80,81]. Electron density shifts (EDSs) were visualized using the Chemcraft program.

## 4. Summary

The calculations reveal that the various σ-hole noncovalent bonds do have certain spectroscopic properties similar to HBs. Just as the X–H bond of the proton donor undergoes an elongation, and its stretching frequency shifts to the red and intensifies, the same occurs for the F–A bond of the Lewis acid. On the other hand, while each of these parameters intensifies as the halogen X atom of the XH proton donor grows larger and the HB becomes weaker, the opposite trend of diminishing spectroscopic change occurs for the other noncovalent bonds with the larger A atom as the bond grows stronger. With respect to NMR chemical shielding, the HBs behave quite differently than do the others. While the bridging H atom is deshielded by HB formation, the other bridging atoms (whether X, Y, Z, or T) all increase their shielding. This shielding change rises quickly along with the size of the particular atom. There is also a strong deshielding noted on the F atoms that lie directly opposite each of these noncovalent bonds. It should be noted that the spectroscopic effects of the formation of these noncovalent bonds upon the Lewis acid when interacting with the NMA surrogate of a peptide bond are qualitatively similar to what was observed recently with the simpler but more powerful NH_3_ base [82].

The spectroscopic manifestations of HBs are much more similar to the other bonds within the Lewis base molecule NMA. In all cases, the C=O bond of the amide is lengthened and its stretching frequency red-shifted and intensified. The amide II band, composed of a C–N stretch and N–H bend, shifts to higher frequency and undergoes a small band weakening. Like the overall bond strength, these effects are heightened by enlarging the bridging A atom. The NMR parameters of all types of bonds are very similar within the base. The shielding of the O atom directly involved in the bond rises by as much as 65 ppm, whereas the C and N atoms of the peptide group both undergo a shielding decrease in the range of 4–8 ppm. These changes rise in magnitude as the bridging A atom grows larger.

Both the frequency shifts of the amide I and II bands of the base as well as the shielding changes of the three pertinent NMA atoms correlate well with the strength of the noncovalent bond. There is thus some hope that spectroscopic measurements can serve not only as a marker for the presence of each of these types of bonds, but also as a gauge for its strength.

## Figures and Tables

**Table 1:** Changes in internal bond lengths (Å), stretching frequencies (cm^−1^), and band intensification resulting^a^ from complex formation.

–	Δr (X–H)	Δν (X–H)	I_comp_/I_mon_	Δr (C=O)	Δν (C=O)^b^	I_comp_/I_mon_	Δν (Amide II Band)^c^	I_comp_/I_mon_
FH∙∙∙NMA	0.032	−729	14.0	0.012	−30	1.6	+26	0.9
ClH∙∙∙NMA	0.051	−695	48.6	0.013	−50	2.8	+25	0.9
BrH∙∙∙NMA	0.060	−705	146.8	0.014	−75	4.8	+26	0.8
IH∙∙∙NMA	0.069	−606	47,960.0	0.014	−169	10.1	+25	1.8
XB	Δr (F−X)	Δν (F−X)						
FCl∙∙∙NMA	0.042	−103	10.5	0.013	−45	2.2	+22	0.9
FBr∙∙∙NMA	0.048	−80	6.8	0.017	−55	2.5	+30	0.8
FI∙∙∙NMA	0.044	−58	4.4	0.019	−55	2.6	+35	0.7
YB	Δr (F−Y)	Δν (F−Y)						
HFS∙∙∙NMA	0.030	−71	3.7	0.009	−28	1.8	+19	1.0
HFSe∙∙∙NMA	0.039	−64	3.4	0.013	−36	2.1	+25	0.9
HFTe∙∙∙NMA	0.041	−56	2.6	0.016	−49	2.3	+30	0.8
ZB	Δr (F–Z)	Δν (F–Z)						
H_2_FP∙∙∙NMA	0.024	−58	2.3	0.008	−24	1.7	+15	1.0
H_2_FAs∙∙∙NMA	0.032	−54	2.2	0.010	−29	1.8	+20	1.0
H_2_FSb∙∙∙NMA	0.036	−51	1.9	0.013	−40	1.9	+25	0.9
TB	Δr (F–T)	Δν (F–T)						
H_3_FSi∙∙∙NMA	0.024	−57	3.2	0.008	−23	1.5	+17	1.0
H_3_FGe∙∙∙NMA	0.031	−57	2.0	0.009	−25	1.6	+20	1.0
H_3_FSn∙∙∙NMA	0.034	−50	1.7	0.013	−35	1.8	+28	0.9

^a^ MP2/aug-cc-pVDZ level; ^b^ amide I band, primarily C=O stretch; ^c^ combination of C–N stretch and N–H bend.

**Table 2:** Changes of NMR chemical shielding (ppm) caused by complexation^a^.

System			Atoms		
HB	H	X	O	C^b^	N
FH∙∙∙NMA	−6.9	−19.5	34.4	−6.3	−5.1
ClH∙∙∙NMA	−9.5	−1.0	20.0	−6.0	−5.4
BrH∙∙∙NMA	−10.6	35.8	15.4	−6.2	−5.6
IH∙∙∙NMA	−11.2	175.7	7.0	−6.1	−5.7
XB	X	F			
FCl∙∙∙NMA	239.3	−139.2	28.4	−5.4	−5.4
FBr∙∙∙NMA	929.2	−208.8	46.5	−7.1	−7.7
FI∙∙∙NMA	2196.7	−296.2	62.6	−7.8	−9.9
YB	Y	F			
HFS∙∙∙NMA	141.2	−112.0	28.1	−4.0	−4.6
HFSe∙∙∙NMA	521.2	−151.9	38.8	−4.9	−6.5
HFTe∙∙∙NMA	1310.8	−193.1	49.4	−7.6	−8.4
ZB	Z	F			
H_2_FP∙∙∙NMA	40.0	−62.2	22.0	−4.0	−4.3
H_2_FAs∙∙∙NMA	107.6	−71.1	27.5	−4.5	−5.5
H_2_FSb∙∙∙NMA	283.9	−80.6	35.2	−6.3	−7.2
TB	T	F			
H_3_FSi∙∙∙NMA	17.3	−42.5	17.0	−4.0	−4.5
H_3_FGe∙∙∙NMA	42.5	−47.3	19.8	−4.3	−5.2
H_3_FSn∙∙∙NMA	127.0	−53.0	29.4	−5.8	−7.6

^a^ MP2 level, aug-cc-pVDZ basis for all atoms except 4th row I, Te, Sb, and Sn, which used all-electron Sapporo-DKH3-DZP-2012-diffuse; ^b^ C atom bonded to O.

**Table 3:** Changes of natural atomic charges (e) caused by complexation^a^

Donors			Atoms		
HB	H	X	O	C	N
FH∙∙∙NMA	−0.003	−0.064	−0.035	0.020	0.025
ClH∙∙∙NMA	0.038	−0.123	−0.013	0.019	0.025
BrH∙∙∙NMA	0.050	−0.155	−0.005	0.020	0.029
IH∙∙∙NMA	0.052	−0.168	0.006	0.020	0.031
XB	X	F			
FCl∙∙∙NMA	−0.072	−0.094	0.044	0.016	0.033
FBr∙∙∙NMA	−0.058	−0.093	0.021	0.019	0.036
FI∙∙∙NMA	−0.050	−0.087	−0.001	0.022	0.040
YB	Y	F			
HFS∙∙∙NMA	−0.043	−0.056	−0.002	0.016	0.023
HFSe∙∙∙NMA	−0.042	−0.063	−0.007	0.016	0.027
HFTe∙∙∙NMA	−0.013	−0.063	−0.023	0.022	0.035
ZB	Z	F			
H_2_FP∙∙∙NMA	−0.035	−0.032	−0.020	0.013	0.016
H_2_FAs∙∙∙NMA	−0.027	−0.039	−0.019	0.014	0.020
H_2_FSb∙∙∙NMA	0.018	−0.046	−0.036	0.021	0.029
TB	T	F			
H_3_FSi∙∙∙NMA	−0.004	−0.025	−0.025	0.015	0.017
H_3_FGe∙∙∙NMA	−0.005	−0.030	−0.025	0.014	0.017
H_3_FSn∙∙∙NMA	−0.001	−0.038	−0.036	0.023	0.032

^a^ MP2 level, aug-cc-pVDZ basis for all atoms except 4th row I, Te, Sb, and Sn, which used all-electron Sapporo-DKH3-DZP-2012-diffuse.

**Table 4:** Interaction energies (E_int_, kcal·mol^−1^) corrected for basis set superposition error (BSSE) calculated at the MP2/aug-cc-pVDZ (I), BLYP-D3/Def2TZVPP (II), and CCSD(T)/aug-cc-pVDZ (III) levels of theory.

System	(I)	(II)	(III)
HB			
FH∙∙∙NMA	−12.68	−14.59	−12.78
ClH∙∙∙NMA	−10.14	−11.27	−9.07
BrH∙∙∙NMA	−9.22	−11.37	−7.80
IH∙∙∙NMA	−7.23	−9.24	−5.38
XB			
FCl∙∙∙NMA	−9.69	−14.88	−8.49
FBr∙∙∙NMA	−13.34	−16.86	−11.92
FI∙∙∙NMA	−15.75	−17.69	−14.40
YB			
HFS∙∙∙NMA	−8.90	−10.32	−8.17
HFSe∙∙∙NMA	−11.18	−12.37	−10.21
HFTe∙∙∙NMA	−13.82	−13.95	−12.89
ZB			
H_2_FP∙∙∙NMA	−7.40	−7.21	−7.00
H_2_FAs∙∙∙NMA	−8.65	−8.53	−8.13
H_2_FSb∙∙∙NMA	−11.40	−10.63	−10.93
TB			
H_3_FSi∙∙∙NMA	−7.70	−7.11	−7.72
H_3_FGe∙∙∙NMA	−8.37	−7.53	−8.34
H_3_FSn∙∙∙NMA	−12.22	−10.20	−12.09

**Table 5:** EDA/BLYP-D3/ZORA/TZ2P decomposition of the interaction energy of complexes into Pauli repulsion (E_Pauli_), electrostatic (E_elec_), orbital interaction (E_oi_), and dispersion (E_disp_) terms. All energies are in kcal/mol. The relative values in percent express the contribution of each to the sum of all attractive energy terms. Geometries are taken from MP2 optimizations.

System	E_int_	E_Pauli_	E_elec_	%	E_oi_	%	E_disp_	%
HB								
FH∙∙∙NMA	−14.86	21.59	−20.70	57	−13.93	38	−1.83	5
ClH∙∙∙NMA	−11.46	28.82	−20.22	50	−17.21	43	−2.85	7
BrH∙∙∙NMA	−11.21	33.58	−21.17	47	−20.34	45	−3.29	7
IH∙∙∙NMA	−8.87	37.65	−21.44	46	−21.53	46	−3.55	8
XB								
FCl∙∙∙NMA	−14.07	32.53	−22.40	48	−21.91	47	−2.29	5
FBr∙∙∙NMA	−17.01	43.71	−31.66	52	−26.58	44	−2.48	4
FI∙∙∙NMA	−17.36	49.65	−37.72	56	−26.66	40	−2.63	4
YB								
HFS∙∙∙NMA	−10.85	25.45	−18.92	52	−14.72	41	−2.66	7
HFSe∙∙∙NMA	−12.75	35.40	−26.27	55	−19.05	40	−2.83	6
HFTe∙∙∙NMA	−13.91	42.18	−32.62	58	−20.26	36	−3.21	6
ZB								
H_2_FP∙∙∙NMA	−8.04	20.43	−15.78	55	−9.91	35	−2.79	10
H_2_FAs∙∙∙NMA	−8.88	26.12	−20.03	57	−11.94	34	−3.02	9
H_2_FSb∙∙∙NMA	−10.59	32.23	−25.76	60	−13.93	33	−3.13	7
TB								
H_3_FSi∙∙∙NMA	−8.32	23.22	−17.73	56	−10.35	33	−3.48	11
H_3_FGe∙∙∙NMA	−7.88	25.96	−19.82	59	−10.51	31	−3.51	10
H_3_FSn∙∙∙NMA	−10.35	35.60	−28.63	62	−14.12	31	−3.20	7

**Table 6:** NBO values (kcal/mol) of E(2) for LP(O) donation to the Lewis acid σ*, orbital sum of all E(2) between NMA and LA, and total charge transfer (CT, in me) from NMA to LA obtained at the BLYP-D3/def2-TVZPP level.

	LP(O)→σ*^a^	Σ(base→acid)	CT
HB			
FH∙∙∙NMA	29.85	31.22	66
ClH∙∙∙NMA	31.24	33.69	84
BrH∙∙∙NMA	39.14	43.25	105
IH∙∙∙NMA	37.36	43.04	107
XB			
FCl∙∙∙NMA	23.94	27.93	167
FBr∙∙∙NMA	26.75	30.63	151
FI∙∙∙NMA	24.80	29.79	122
YB			
HFS∙∙∙NMA	13.05	16.21	86
HFSe∙∙∙NMA	17.12	22.03	93
HFTe∙∙∙NMA	17.65	23.28	80
ZB			
H_2_FP∙∙∙NMA	6.03	8.24	43
H_2_FAs∙∙∙NMA	10.41	13.32	54
H_2_FSb∙∙∙NMA	11.34	16.21	57
TB			
H_3_FSi∙∙∙NMA	5.24	10.63	41
H_3_FGe∙∙∙NMA	7.30	12.78	41
H_3_FSn∙∙∙NMA	9.37	19.21	56

^a^ Sum of O lone pair transfers to σ*(H–X) for HB and σ*(A–F) for others.

## Supplementary Materials

The supplementary materials are available online. Figure S1: Electron Density Shifts calculated at the MP2. Purple indicates gain and loss is shown in green. Contours of Δρ = ±0.0005 au on left and ±0.0015 au. on right, Table S1: NMR chemical shielding (ppm) of isolated monomers at MP2 level, Table S2: NMR chemical shielding (ppm) of isolated NMA molecule at MP2/aug-cc-pVDZ level, Table S3: NMR chemical shielding (ppm) for complexes at MP2 level, Table S4: AIM descriptors of complexes studied. Bond critical point (BCP) properties: electron density *ρ*, Laplacian of electron density ∇^2^*ρ*, ellipticity ε, and total electron energy H, were obtained at the MP2/aug-cc-pVDZ level. Data is given in atomic units, Table S5: Coordinates of complexes optimized at MP2/aug-cc-pVDZ level.
