# Supplementary material for: Theoretical Studies of IR and NMR Spectral Changes Induced by Sigma-Hole Hydrogen, Halogen, Chalcogen, Pnicogen, and Tetrel Bonds in a Model Protein Environment
Source: Molecules. 2019 Sep 12;24(18):3329. doi: 10.3390/molecules24183329 (PMC6767630; doi:10.3390/molecules24183329)
Supplement: Supplementary file 1 [file molecules-24-03329-s001.pdf]

molecules

MDPI

*Supplementary Materials*

# IR and NMR Spectral Changes Induced by Halogen, Chalcogen, Pnicogen, and Tetrel Bonds in a Model Protein Environment

**Mariusz Michalczyk [1], Wiktor Zierkiewicz [1,*], Rafał Wysokiński [1] and Steve Scheiner [2,*]**

[1] Faculty of Chemistry, Wrocław University of Science and Technology, Wybrzeże Wyspiańskiego 27, 50-370 Wrocław, Poland

[2] Department of Chemistry and Biochemistry, Utah State University, Logan, UT 84322-0300, USA

* Correspondence: wiktor.zierkiewicz@pwr.edu.pl (W.Z.); steve.scheiner@usu.edu (S.S.)

**Table S1.** NMR chemical shielding (ppm) of isolated monomers at the MP2/aug-cc-pVDZ level.

| Monomer | Atoms | |
|---|---|---|
| HB | X | H |
| HF | 428.0 | 29.6 |
| HCl | 995.0 | 30.9 |
| HBr | 2729.0 | 31.3 |
| HI | 460.4 | 31.7 |
| XB | X | F |
| ClF | -430.6 | 635.7 |
| BrF | -568.7 | 766.1 |
| IF | 311.73 | 928.7 |
| YB | Y | F |
| SHF | -90.2 | 654.3 |
| SeHF | 317.4 | 695.2 |
| TeHF | 330.0 | 752.9 |
| ZB | Z | F |
| $PH_2F$ | 328.2 | 526.9 |
| $AsH_2F$ | 1409.5 | 535.6 |
| $SbH_2F$ | 348.4 | 539.0 |
| TB | T | F |
| $SiH_3F$ | 463.5 | 442.3 |
| $GeH_3F$ | 1683.0 | 471.7 |
| $SnH_3F$ | 307.2 | 487.9 |

**Table S2.** NMR chemical shielding (ppm) of the isolated NMA molecule at the MP2/aug-cc-pVDZ level.

| | O | C | N |
|---|---|---|---|
| NMA | -2.1 | 43.3 | 176.5 |

**Table S3.** NMR chemical shielding (ppm) for complexes at the MP2/aug-cc-pVDZ level.

| System | Atoms | | | | |
|---|---|---|---|---|---|
| HB | H | X | O | C | N |
| FH···NMA | 22.7 | 408.5 | 32.3 | 37.0 | 171.4 |
| ClH···NMA | 21.4 | 994.0 | 17.9 | 37.3 | 171.1 |
| BrH···NMA | 20.7 | 2764.8 | 13.3 | 37.1 | 170.9 |
| IH···NMA | 20.4 | 466.4 | 5.0 | 37.2 | 170.9 |
| XB | X | F | | | |
| FCl···NMA | -191.3 | 496.5 | 26.3 | 37.9 | 171.1 |
| FBr···NMA | 360.5 | 557.3 | 44.4 | 36.2 | 168.8 |
| FI···NMA | 358.7 | 646.4 | 63.2 | 35.5 | 166.6 |
| YB | Y | F | | | |
| HFS···NMA | 51.0 | 542.3 | 26.0 | 39.3 | 171.9 |
| HFSe···NMA | 838.6 | 543.3 | 36.7 | 38.4 | 170.0 |
| HFTe···NMA | 355.9 | 564.2 | 50.4 | 35.6 | 168.0 |
| ZB | Z | F | | | |
| $H_2FP$···NMA | 368.2 | 464.7 | 19.9 | 39.3 | 172.2 |
| $H_2FAs$···NMA | 1517.1 | 464.5 | 25.4 | 38.8 | 171.0 |
| $H_2FSb$···NMA | 351.4 | 461.2 | 35.2 | 36.9 | 169.2 |
| TB | T | F | | | |
| $H_3FSi$···NMA | 480.8 | 399.8 | 14.9 | 39.3 | 172.0 |
| $H_3FGe$···NMA | 1725.5 | 424.4 | 17.7 | 39.0 | 171.3 |
| $H_3FSn$···NMA | 306.5 | 434.9 | 26.3 | 37.5 | 169.0 |

[a] MP2/aug-cc-pVDZ level.

**Table S4.** AIM descriptors of complexes studied. Bond critical point (BCP) properties: electron density $\rho$, Laplacian of the electron density $\nabla^2\rho$, ellipticity ε, and total electron energy H, were obtained at the MP2/aug-cc-pVDZ level. Data are given in atomic units.

| | Interaction | $\rho$ | $\nabla^2\rho$ | ε | H |
|---|---|---|---|---|---|
| | | | HB | | |
| FH···NMA | H···O | 0.052 | 0.173 | 0.010 | -0.005 |
| ClH···NMA | H···O | 0.054 | 0.143 | 0.013 | -0.006 |
| BrH···NMA | H···O | 0.057 | 0.142 | 0.014 | -0.007 |
| IH···NMA | H···O | 0.058 | 0.136 | 0.014 | -0.008 |
| | | | XB | | |
| FCl···NMA | Cl···O | 0.043 | 0.146 | 0.019 | -0.001 |
| FBr···NMA | Br···O | 0.049 | 0.144 | 0.018 | -0.004 |
| FI···NMA | I···O | 0.046 | 0.132 | 0.015 | -0.006 |
| | | | YB | | |
| HFS···NMA | S···O | 0.032 | 0.099 | 0.180 | -0.001 |
| HFSe···NMA | Se···O | 0.039 | 0.108 | 0.260 | -0.004 |
| HFTe···NMA | Te···O | 0.039 | 0.111 | 0.234 | -0.004 |
| | | | ZB | | |
| $H_2$FP···NMA | P···O | 0.025 | 0.064 | 0.019 | -0.002 |
| $H_2$FAs···NMA | As···O | 0.029 | 0.077 | 0.035 | -0.003 |
| $H_2$FSb···NMA | Sb···O | 0.030 | 0.090 | 0.058 | -0.002 |
| | | | TB | | |
| $H_3$FSi···NMA | Si···O | 0.022 | 0.052 | 0.417 | -0.003 |
| $H_3$FGe···NMA | Ge···O | 0.025 | 0.081 | 0.018 | -0.002 |
| $H_3$FSn···NMA | Sn···O | 0.032 | 0.106 | 0.009 | -0.002 |

| | 0.0005 | 0.0015 |
|---|---|---|
| HB | | |
| FH···NMA | 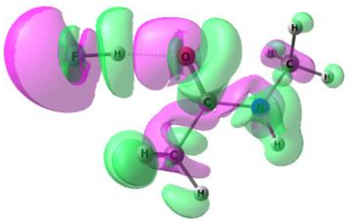 | 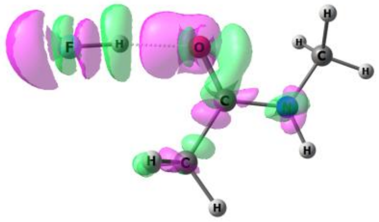 |
| ClH···NMA | 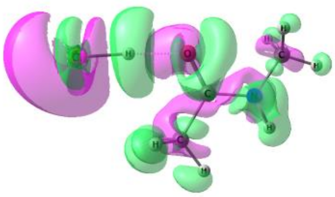 | 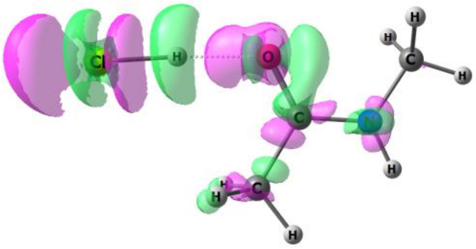 |
| BrH···NMA | 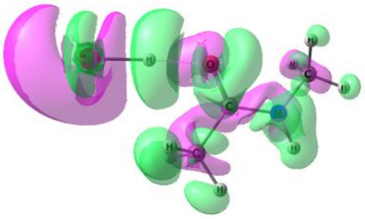 | 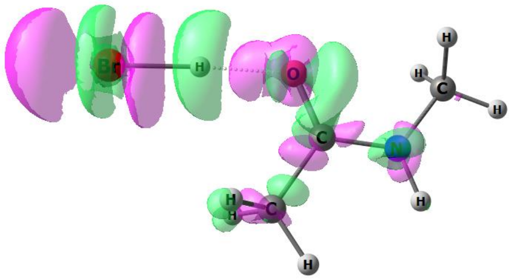 |
| IH···NMA | 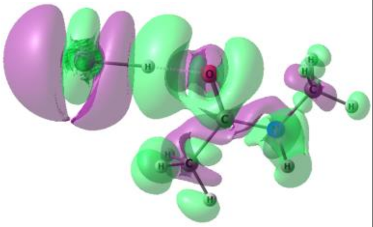 | 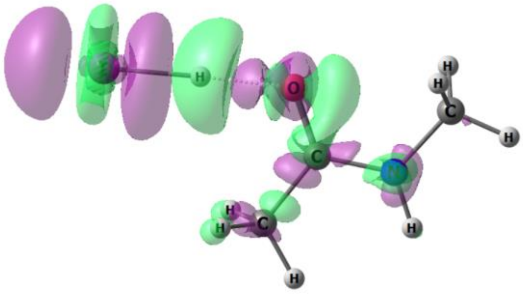 |
| XB | | |
| FCl···NMA | 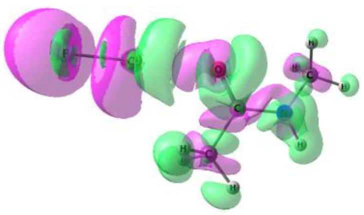 | 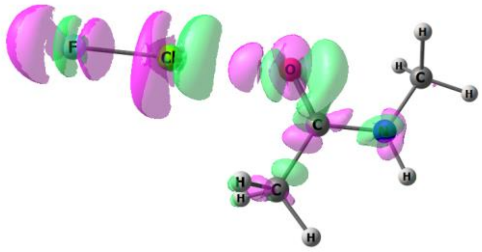 |

| | | |
|---|---|---|
| FBr···NMA | 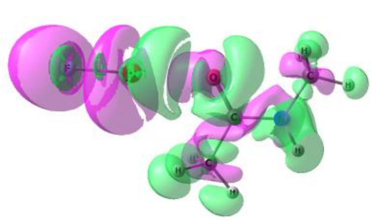 | 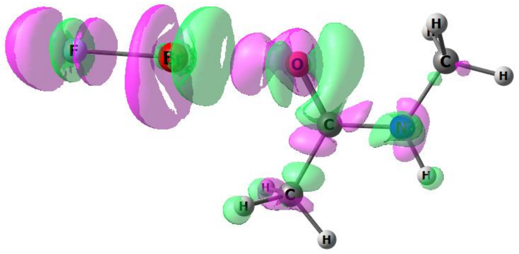 |
| FI···NMA | 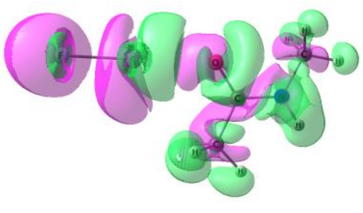 | 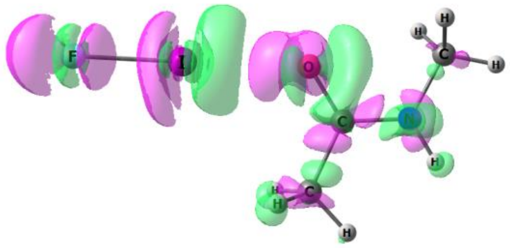 |
| YB | | |
| HFS···NMA | 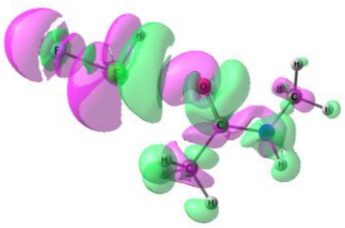 | 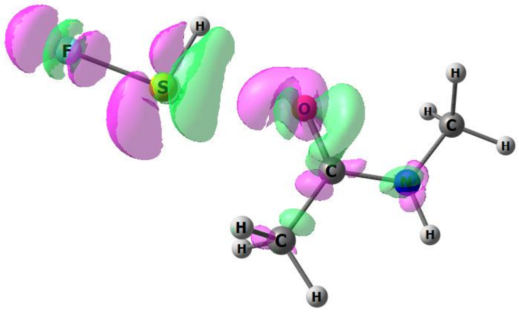 |
| HFSe···NM<br>A | 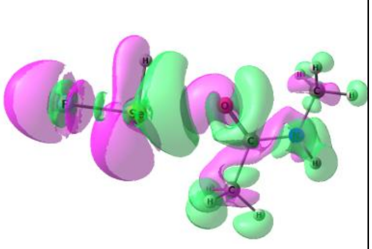 | 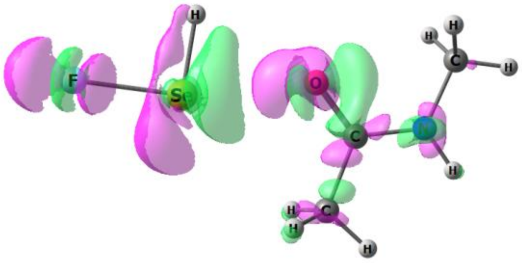 |
| HFTe···NM<br>A | 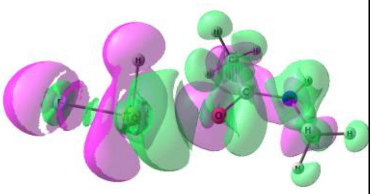 | 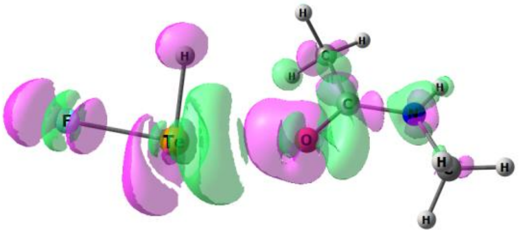 |
| ZB | | |
| $H_2FP$···NM<br>A | 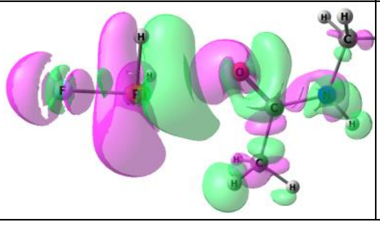 | 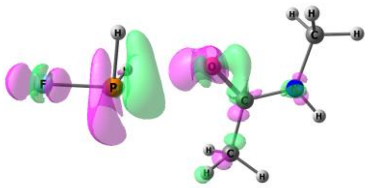 |

| | | |
|---|---|---|
| $H_2FAs$···NMA | 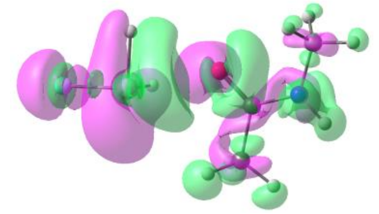 | 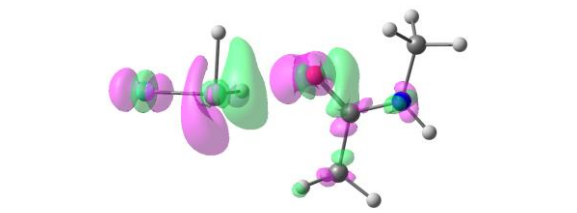 |
| $H_2FSb$···NMA | 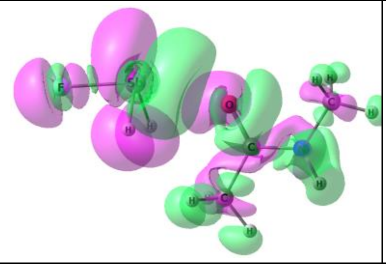 | 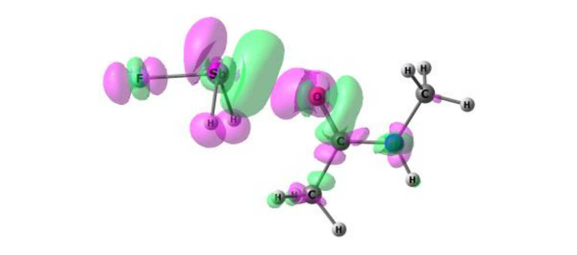 |
| TB | | |
| $H_3FSi$···NMA | 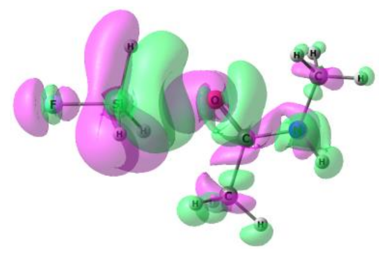 | 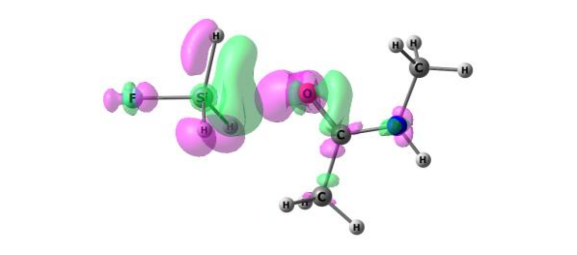 |
| $H_3FGe$···NMA | 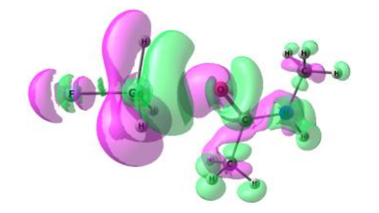 | 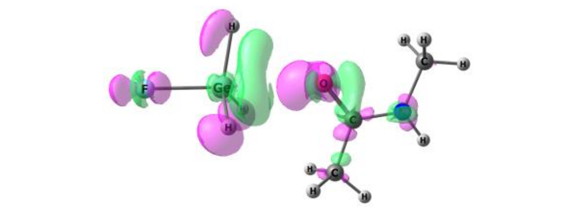 |
| $H_3FSn$···NMA | 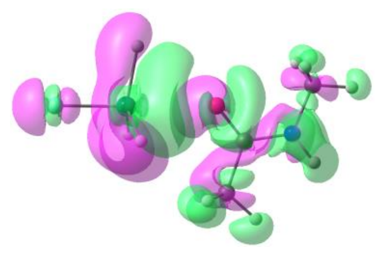 | 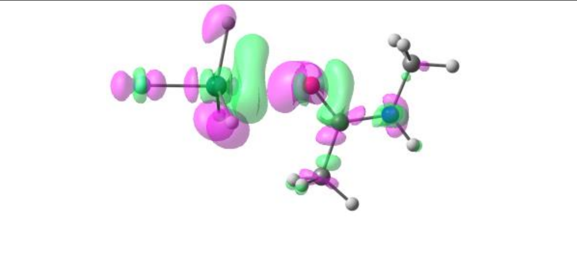 |

**Figure S1.** Electron density shifts calculated at the MP2/aug-cc-pVDZ level. Purple indicates gain and loss is shown in green. Contours of $\Delta\rho = \pm0.0005$ au on left and ±0.0015 au. on right.

**Table S5.** Coordinates of complexes optimized at the MP2/aug-cc-pVDZ level.

| System | Coordinates |
|---|---|
| HB | |
| FH···NMA | C -1.52710000 1.23369400 0.00493700<br>C -0.77809500 -0.08012800 -0.01091300<br>H -1.05591600 -0.65589100 0.88361600<br>H -1.10035600 -0.65985200 -0.88780300<br>H 0.31236200 0.05618900 -0.03857600<br>H 0.21740300 2.30287700 -0.04095800<br>C -1.43734700 3.68125600 -0.00259900<br>H -2.04848500 3.79576600 0.90417600 |

| | |
|---|---|
| | H -0.65899500 4.45440600 -0.02376800 |
| | H -2.09316600 3.79196400 -0.87808900 |
| | N -0.79094800 2.37168500 -0.01602600 |
| | O -2.77526200 1.29509400 0.03606900 |
| | H -3.51846700 -0.13316800 0.05785100 |
| | F -3.86570500 -1.02530600 0.06854300 |
| ClH···NMA | C -1.51083800 1.25814100 0.00508000 |
| | C -0.77120800 -0.06133900 -0.00876800 |
| | H -1.05430300 -0.63715600 0.88419000 |
| | H -1.09150900 -0.63930900 -0.88768500 |
| | H 0.32015700 0.06912200 -0.03191200 |
| | H 0.24348500 2.31128700 -0.03709400 |
| | C -1.39862400 3.70508000 -0.00349300 |
| | H -2.01100300 3.82550800 0.90165900 |
| | H -0.61269800 4.47054100 -0.02287700 |
| | H -2.05091000 3.82135200 -0.88088700 |
| | N -0.76448300 2.38930800 -0.01461400 |
| | O -2.75932400 1.33161500 0.03323900 |
| | H -3.54617200 -0.10138400 0.05277400 |
| | Cl -4.11264800 -1.31417800 0.06684700 |
| BrH···NMA | C -1.51111900 1.26612200 0.00551500 |
| | C -0.78315600 -0.05960900 -0.00864900 |
| | H -1.07072700 -0.63385200 0.88388000 |
| | H -1.10836500 -0.63553800 -0.88708900 |
| | H 0.30909900 0.06313600 -0.03200800 |
| | H 0.25169800 2.30301300 -0.03789300 |
| | C -1.37783200 3.71190900 -0.00340400 |
| | H -1.98790800 3.83876200 0.90240700 |
| | H -0.58500900 4.47014300 -0.02451100 |
| | H -2.02999500 3.83295400 -0.88022400 |
| | N -0.75558000 2.39038300 -0.01381500 |
| | O -2.75981200 1.35282100 0.03394100 |
| | H -3.53917300 -0.06671100 0.05198700 |
| | Br -4.17219900 -1.40494600 0.06632400 |
| IH···NMA | C -1.50115800 1.27814300 0.00449400 |
| | C -0.76443800 -0.04269300 -0.01127700 |
| | H -1.04364100 -0.61823400 0.88313800 |
| | H -1.08804700 -0.62193300 -0.88817500 |
| | H 0.32680300 0.08822900 -0.03891900 |
| | H 0.25543200 2.32526800 -0.04206800 |
| | C -1.38239500 3.72469200 -0.00405700 |
| | H -1.99174100 3.84688600 0.90290800 |
| | H -0.59408200 4.48760800 -0.02572200 |

| | |
|---|---|
| | H -2.03672100 3.84274800 -0.87969100<br>N -0.75240800 2.40679600 -0.01688600<br>O -2.75061300 1.35800800 0.03540600<br>H -3.52323700 -0.07253600 0.05763300<br>I -4.27383000 -1.57439500 0.07967700 |
| XB | |
| FCl···NMA | C -1.42373200 1.34473500 0.00272600<br>C -0.72565900 0.00373800 -0.00910600<br>H -1.02669300 -0.56102900 0.88476600<br>H -1.06166800 -0.56359200 -0.88878400<br>H 0.36909800 0.10245100 -0.03083500<br>H 0.36501600 2.34059600 -0.03469700<br>C -1.23063800 3.78738200 -0.00513900<br>H -1.84086100 3.92840700 0.89860100<br>H -0.42012900 4.52683100 -0.02289500<br>H -1.87685300 3.92500300 -0.88404400<br>N -0.63986100 2.45181500 -0.01453100<br>O -2.66865700 1.46495400 0.02732500<br>Cl -3.97735600 -0.45673500 0.05585500<br>F -4.96208500 -1.86597100 0.07721900 |
| FBr···NMA | C -1.39669700 1.34409400 0.00204000<br>C -0.66974000 0.02148300 -0.01047100<br>H -0.95899400 -0.55133800 0.88223900<br>H -0.99350500 -0.55355700 -0.88980000<br>H 0.42191200 0.14847000 -0.03188400<br>H 0.35958200 2.38694700 -0.03333300<br>C -1.27629700 3.79013100 -0.00324300<br>H -1.89042000 3.91154000 0.90048700<br>H -0.48624200 4.55114700 -0.01950700<br>H -1.92505900 3.90938800 -0.88273100<br>N -0.64818800 2.47080300 -0.01385400<br>O -2.64794400 1.44036800 0.02622900<br>Br -3.97000300 -0.47099700 0.05397400<br>F -5.03848100 -1.96989300 0.07631400 |
| FI···NMA | C -1.96787200 0.46003500 0.00011700<br>C -1.55283800 1.90949000 -0.00000200<br>H -0.92895600 2.09937900 0.88528000<br>H -0.92883900 2.09921700 -0.88523500<br>H -2.41669200 2.58919600 -0.00010900<br>H -3.94872500 0.94911200 -0.00068200<br>C -3.76732900 -1.19943200 -0.00025400<br>H -3.40153400 -1.72731300 0.89192200<br>H -4.86426900 -1.18717000 -0.00078500 |

| | |
|---|---|
| | H -3.40062900 -1.72738400 -0.89201800<br>N -3.28947300 0.18196600 -0.00006200<br>O -1.14507800 -0.48988900 0.00045800<br>I 1.25731800 -0.15603600 0.00005000<br>F 3.24077500 0.08884900 -0.00038100 |
| YB | |
| HFS···NMA | C -1.25643500 1.26896400 0.05047900<br>C -0.43313000 0.00401800 0.14894400<br>H -0.69258800 -0.51235000 1.08479800<br>H -0.70524900 -0.65839200 -0.68589100<br>H 0.64863800 0.19882600 0.12514800<br>H 0.43162500 2.42739700 -0.05769300<br>C -1.29426900 3.71118700 -0.15119600<br>H -1.92645200 3.86481600 0.73543900<br>H -0.55831900 4.52194500 -0.22370000<br>H -1.93961400 3.71784800 -1.04162400<br>N -0.57927900 2.44252000 -0.05157700<br>O -2.50372500 1.25952100 0.06047300<br>F -4.82341600 -2.14491100 0.35841900<br>H -4.60032100 0.08621400 0.17255100<br>S -3.67388900 -0.88725600 0.24623500 |
| HFSe···NMA | C -1.22975800 1.26691600 0.07539300<br>C -0.38315700 0.02963300 0.26023700<br>H -0.68914400 -0.46991500 1.19156100<br>H -0.58289700 -0.66076400 -0.57283500<br>H 0.69196400 0.25466000 0.30062600<br>H 0.42126900 2.47541500 0.04461000<br>C -1.33593800 3.69899000 -0.19378700<br>H -2.02861500 3.85126200 0.64646500<br>H -0.62287700 4.53157700 -0.24020900<br>H -1.92138600 3.66080800 -1.12373300<br>N -0.58805000 2.45736500 -0.01489600<br>O -2.47763000 1.22294500 0.00497000<br>F -4.85425300 -2.24738600 0.23678000<br>H -4.67510000 0.11885700 -0.00831900<br>Se -3.63085000 -0.89001800 0.16394200 |
| HFTe···NMA | C -2.01848800 0.47549200 -0.01127900<br>C -1.64278700 1.93598100 0.04604000<br>H -0.87475900 2.07298700 0.82078900<br>H -1.19513900 2.22607600 -0.91654600<br>H -2.50790800 2.58198500 0.25282300<br>H -4.01105000 0.91514500 0.08928300<br>C -3.78155600 -1.22353400 -0.04772300 |

| | |
|---|---|
| | H -3.37560000 -1.79703500 0.79777700<br>H -4.87797500 -1.23822100 -0.01263500<br>H -3.43304400 -1.68764900 -0.98137700<br>N -3.33471600 0.16600400 0.02309800<br>O -1.17158000 -0.44547500 -0.10031200<br>F 3.23157700 0.22357800 -0.14923700<br>H 0.95614600 0.71686400 -1.26573700<br>Te 1.30014900 -0.20246600 0.06302600 |
| ZB | |
| $H_2FP$···NMA | C -1.30590400 1.35954100 0.00819500<br>C -0.58705000 0.02792300 -0.03268700<br>H -0.90259400 -0.56858200 0.83505900<br>H -0.89791700 -0.51341600 -0.93853500<br>H 0.50728800 0.13560300 -0.03252900<br>H 0.47389200 2.37936200 -0.05826000<br>C -1.13758800 3.80479000 0.01495200<br>H -1.71344400 3.94083800 0.94207500<br>H -0.33845700 4.55527600 -0.03548100<br>H -1.82025200 3.93465500 -0.83728500<br>N -0.53157200 2.47747500 -0.01928000<br>O -2.54750600 1.45171500 0.05887100<br>H -4.23411300 -0.32172100 -0.81846400<br>H -4.76267700 0.61150300 0.96113900<br>P -4.06016800 -0.56440200 0.57513800<br>F -5.38130600 -1.61789200 0.73258700 |
| $H_2FAs$···NMA | C -1.31024800 1.37273600 0.04297800<br>C -0.61578600 0.03205600 -0.05422400<br>H -0.83713500 -0.54525100 0.85551400<br>H -1.03055600 -0.52024700 -0.91045100<br>H 0.47294300 0.12512100 -0.17541200<br>H 0.46829700 2.37273800 -0.15267200<br>C -1.11834800 3.81480100 0.05088600<br>H -1.61539600 3.95165600 1.02230100<br>H -0.31818300 4.55714500 -0.06188200<br>H -1.86635400 3.95350900 -0.74290100<br>N -0.53036200 2.48121300 -0.03735700<br>O -2.54498200 1.48050000 0.18827900<br>H -3.98429000 -0.50298900 -0.83550000<br>H -4.94870300 0.71077200 0.73564900<br>As -4.04155300 -0.50241000 0.68404700<br>F -5.41871100 -1.68868200 0.74623900 |
| $H_2FSb$···NMA | C -2.08759400 0.48724500 0.00009800<br>C -1.74004700 1.95747600 -0.00013000 |

| | |
|---|---|
| | H -1.12394900 2.17602100 0.88419400<br>H -1.13055000 2.17728800 -0.88874900<br>H -2.63143100 2.60078700 0.00351600<br>H -4.09168500 0.90062000 0.00075300<br>C -3.82729900 -1.23715200 0.00003900<br>H -3.44317200 -1.75253200 0.89207300<br>H -4.92406300 -1.26795100 -0.00110100<br>H -3.44130300 -1.75260200 -0.89113200<br>N -3.40267100 0.16039500 0.00028000<br>O -1.22155800 -0.41570000 -0.00017200<br>H 0.93060200 0.84169700 1.22889500<br>H 0.93068200 0.84345000 -1.22755000<br>Sb 1.35921000 -0.26316400 -0.00010900<br>F 3.23621600 0.40133400 0.00044500 |
| TB | |
| $H_3FSi\cdots NMA$ | C -1.41766800 1.27934800 0.00365900<br>C -0.65563700 -0.02854900 0.00182200<br>H -0.93930100 -0.60450700 0.89468100<br>H -0.95405100 -0.61060000 -0.88228200<br>H 0.43428000 0.11481300 -0.00783900<br>H 0.32793300 2.35662400 -0.02623900<br>C -1.33201200 3.72801800 -0.01400000<br>H -1.95585500 3.84488100 0.88399800<br>H -0.55665200 4.50444100 -0.02797000<br>H -1.97689300 3.83380700 -0.89842500<br>N -0.68093800 2.42137200 -0.01340400<br>O -2.66292800 1.33231100 0.01965300<br>H -3.48371700 -0.83506000 1.27997600<br>H -3.49951500 -0.84905700 -1.20566100<br>H -5.11672500 0.59023000 0.03904700<br>F -5.36978400 -1.89060600 0.05491200<br>Si -4.26874200 -0.61920500 0.04086300 |
| $H_3FGe\cdots NMA$ | C -1.40959300 1.28969400 0.00406100<br>C -0.65628800 -0.02294300 0.00280300<br>H -0.94422200 -0.59688700 0.89570100<br>H -0.95834100 -0.60326900 -0.88131000<br>H 0.43451500 0.11282400 -0.00648000<br>H 0.34004200 2.35773000 -0.02602800<br>C -1.31391500 3.73710300 -0.01447600<br>H -1.93685800 3.85670600 0.88370700<br>H -0.53507900 4.50991500 -0.02902000<br>H -1.95832100 3.84476800 -0.89893700<br>N -0.66858600 2.42729900 -0.01335100 |

| | |
|---|---|
| | O -2.65540700 1.34894300 0.01999000 |
| | H -3.48004800 -0.86037700 1.32682200 |
| | H -3.49468200 -0.87449600 -1.25277600 |
| | H -5.17774200 0.61252500 0.03813800 |
| | F -5.43239400 -1.96938200 0.05393800 |
| | Ge -4.26128900 -0.60189300 0.04000500 |
| $H_3FSn$···NMA | C -2.06187600 0.46094400 0.00154700 |
| | C -1.73388800 1.93616600 0.00009000 |
| | H -1.12730500 2.16431100 0.88891800 |
| | H -1.12700200 2.16253000 -0.88900600 |
| | H -2.63227400 2.56933000 -0.00075800 |
| | H -4.07074000 0.84320300 -0.00689400 |
| | C -3.77342600 -1.29074600 -0.00238400 |
| | H -3.38315000 -1.79850100 0.89119800 |
| | H -4.86947000 -1.33800800 -0.00557100 |
| | H -3.37784800 -1.80081400 -0.89232400 |
| | N -3.37029200 0.11364300 -0.00289000 |
| | O -1.17983100 -0.42648600 0.00670100 |
| | H 0.98640700 0.69153200 1.44521400 |
| | H 0.97837200 0.69704900 -1.43920800 |
| | H 1.19987500 -1.82380000 -0.00322900 |
| | F 3.29921400 0.05325400 -0.00482100 |
| | Sn 1.32352100 -0.13735800 0.00052300 |
